# The Role of Sodium Glucose Cotransporter-2 Inhibitors in Atherosclerotic Cardiovascular Disease: A Narrative Review of Potential Mechanisms

**DOI:** 10.3390/cells10102699

**Published:** 2021-10-09

**Authors:** Jennifer Y. Barraclough, Sanjay Patel, Jie Yu, Bruce Neal, Clare Arnott

**Affiliations:** 1 The George Institute for Global Health, University of New South Wales, Sydney, NSW 2042, Australia; jbarraclough@georgeinstitute.org.au (J.Y.B.); Jyu1@georgeinstitute.org.au (J.Y.); bneal@georgeinstitute.org.au (B.N.); 2 Department of Cardiology, Royal Prince Alfred Hospital, Sydney, NSW 2050, Australia; Sanjay.Patel2@health.nsw.gov.au; 3 Sydney Medical School, University of Sydney, Sydney, NSW 2042, Australia

**Keywords:** sodium glucose cotransporter 2 inhibitor, atherosclerosis, cardiovascular disease, inflammation, mechanism of action

## Abstract

Sodium glucose cotransporter 2 (SGLT2) inhibitors are a class of medication with broad cardiovascular benefits in those with type 2 diabetes, chronic kidney disease, and heart failure. These include reductions in major adverse cardiac events and cardiovascular death. The mechanisms that underlie their benefits in atherosclerotic cardiovascular disease (ASCVD) are not well understood, but they extend beyond glucose lowering. This narrative review summarises the ASCVD benefits of SGLT2 inhibitors seen in large human outcome trials, as well as the mechanisms of action explored in rodent and small human studies. Potential pathways include favourable alterations in lipid metabolism, inflammation, and endothelial function. These all require further investigation in large human clinical trials with mechanistic endpoints, to further elucidate the disease modifying benefits of this drug class and those who will benefit most from it.

## 1. Introduction

Sodium glucose cotransporter 2 (SGLT2) inhibitors are a class of medication that act in the proximal nephron to reduce glucose reabsorption, thereby causing glycosuria and modest reductions in blood sugar levels. They entered the market initially as an oral hypoglycaemic for use in people with type 2 diabetes (T2D), with canagliflozin being the first to obtain U.S. Food and Drug Administration (FDA) approval in 2013 [1].

Several large scale clinical trials, including EMPA-REG Outcome [2] (empagliflozin in those with T2D and established cardiovascular (CV) disease), the CANVAS Program [1] (canagliflozin in those with T2D and either established CV disease or high risk for CV disease), DECLARE-TIMI 58 [3] (dapagliflozin in those with T2D and either established CV disease or high risk for CV disease), and CREDENCE [4] (canagliflozin in those with both T2D and diabetic kidney disease) have demonstrated significant CV and renal benefits for this drug class. These include proportional reductions of more than 30% for hospitalisation for heart failure (HHF), 15% for all-cause mortality, 17% for CV mortality [5], and 30% for dialysis, transplantation, or death due to kidney disease [6]. The role of SGLT2 inhibitors in reducing cardiovascular events attributable to atherosclerotic cardiovascular disease (ASCVD), however, has been questioned, due to inconclusive results with respect to myocardial infarction (MI) and stroke outcomes. Meta-analyses suggest this drug class reduces major adverse cardiovascular events (MACE) and some of its components, including fatal/non-fatal myocardial infarction, by 12% [5]. However, there is heterogeneity in the individual clinical trials with respect to MI outcomes, particularly in those without established CV disease. The data on strokes are of particular interest, with little evidence that SGLT2 inhibitors reduce the incidence of fatal or non-fatal stroke, despite clear effects on blood pressure [5]. The recently published SCORED trial is the only study to demonstrate a reduction in stroke from SGLT2 inhibition, though that was only identified in a post hoc secondary analysis (HR 0.66, 95% CI 0.48 to 0.91) [7]. A possible signal of reduction in stroke in those with reduced kidney function identified in a recent meta-analysis has raised additional questions about how the drug class might be effecting mechanisms of atherosclerosis [5].

This narrative review consolidates the available literature from animal and human studies describing the major clinical outcomes of SGLT2 inhibition in ASCVD and explores the potential mechanisms underpinning these effects with key findings presented.

## 2. Large Scale Clinical Trial Outcomes

To date, there have been six event-driven randomised placebo control trials of SGLT2 inhibition undertaken in T2D populations: the EMPA-REG Outcome trial [2], the CANVAS Program [1] (CANVAS and CANVAS-R), the DECLARE-TIMI58 trial [3], the CREDENCE trial [4], the VERTIS trial [8], and the SCORED trial [7]. One study, DAPA-CKD [9], was conducted in patients with chronic kidney disease (CKD), irrespective of T2D status, whilst CREDENCE [4] and SCORED [7] recruited those with both T2D and CKD. Two studies, DAPA-HF [10] and EMPORER-Reduced [11], were conducted in patients with heart failure with reduced ejection fraction (HFrEF). However, 41.8% of participants in DAPA-HF [10] and 49.8% in EMPORER-Reduced [11] had T2D. The proportion of individuals with established ASCVD in each trial is outlined in Table 1 and ranges from 40.6% in DECLARE-TIMI to 100% in EMPA-REG Outcome [2] and VERTIS [8].

In those with T2D, a recent meta-analysis (including EMPA-REG Outcome [2], CANVAS Program [1], DECLARE-TIMI58 [3] and CREDENCE [4]) reported an overall significant reduction in MACE in those treated with SGLT2 inhibition as compared to placebo (HR 0.88, 95% CI 0.82 to 0.94). There was no evidence that this treatment effect differed by baseline history of ASCVD in the study participants (*p* heterogeneity = 0.252), although the outcome did not reach separate statistical significance in those without a history of ASCVD (HR 0.94, 95% CI 0.82 to 1.07) [5]. This likely reflects the relatively small number of events that occurred in the primary prevention group rather than a true lack of efficacy in this group. These results are supported by contributing trials, with CANVAS [1] (HR 0.86, 95% CI 0.75 to 0.97), EMPA-REG Outcome [2] (HR 0.86, CI 0.74 to 0.99), CREDENCE [4] (HR 0.80, 95% CI 0.67 to 0.95), and SCORED [7] (HR 0.84, 95% CI 0.72 to 0.99), all reporting a significant reduction in MACE with SGLT2 inhibition. DECLARE-TIMI [3] and VERTIS-CV [8] did not demonstrate a statistically significant reduction in MACE, but both reported hazard ratios less than 1 for this outcome. (Table 1)

With respect to MI, the meta-analysis suggests a 12% reduction (HR 0.88, 95% CI 0.80 to 0.97) with SGLT2 inhibition, though no individual studies achieved statistical significance for this outcome [5] apart from SCORED, which reported a reduction of 32% (HR 0.68, 95% CI 0.52 to 0.89) [7,12]. The same is true for analyses done comparing subgroups defined by history of ASCVD at baseline, where there was no evidence of different effects detected, though limited statistical power to address this question.

Substantial reductions in CV mortality are clear when analysing the aggregate data (HR 0.83, 95% CI 0.75 to 0.92) and there were early indications of possible large drug-specific differences in effect for this outcome [5]. This was consequent upon a significant disparity between the CV mortality data for the first two trials to report, EMPA-REG Outcome (HR 0.62, 95% CI 0.49 to 0.77) and the CANVAS Program (HR 0.87, 95% CI 0.72 to 1.06). It was postulated that this observation might reflect greater effects amongst patients with a history of ASCVD (100% in EMPA-REG outcome versus 66% in the CANVAS Program), but subsequent investigations from large meta-analyses comparing effects in those with and without baseline ASCVD fail to identify any difference in effects between these participant subgroups (p heterogeneity = 0.167) [5]. In addition the large effect of empagliflozin observed in EMPA-REG Outcome was not repeated in EMPEROR-reduced (HR 0.92, 95%CI 0.75 to 1.12) [11]; thus, the magnitude of the EMPA-REG Outcome results for CV mortality (and total mortality) were likely chance findings. Furthermore, more recent aggregate data inclusive of Sotagliflozin, a SGLT1 and 2 inhibitor, demonstrate very similar CV mortality benefits (HR 0.84, 95%CI 0.74–0.96) [12].

Results in the CKD population largely reflect those seen in the T2D studies. In DAPA-CKD [9], SGLT2 inhibition results in a 19% reduction in CV mortality (HR 0.81, 95% CI 0.58 to 1.12) and similarly, in the heart failure population DAPA-HF [10] demonstrated a CV mortality benefit from SGLT2 inhibition of 18% (HR 0.82, 95% CI 0.69 to 0.98). 

These clinical trials have also demonstrated consistent benefits for this drug class on intermediate markers of cardiovascular risk. In particular, significant reductions in body weight, blood pressure, albuminuria, and glycosylated haemoglobin (HbA1C) were observed [1,2,3,4]. This offers a potential mechanism by which SGLT2 inhibitors could be mediating an ASCVD benefit in treated individuals. Whilst contributory, it is unlikely however that these changes alone are responsible for the ASCVD benefits identified in these clinical trials. This is certainly true when assessing the heart failure benefit of SGLT2 inhibition. Mediation analyses suggest that changes in systolic blood pressure, HbA1C, and body weight only contribute a small percentage of the overall benefit with respect to hospitalization for heart failure [13].

These clinical benefits, however, should be considered within the context of the broader safety profile. Indeed, whilst this drug class is associated with a reduction in total serious adverse events, there is an increased risk of ketoacidosis and genital mycotic infections [5].

## 3. The Pathophysiology of Atherosclerosis

**Figure 1:**
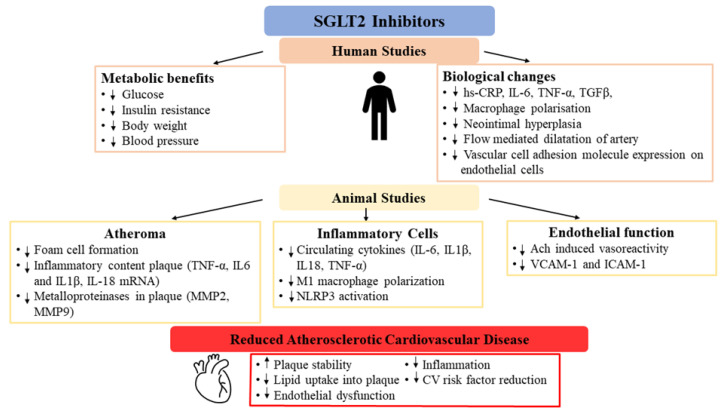
Mechanisms of action of SGLT2 inhibitors in atherosclerotic cardiovascular disease; Ach—acetylcholine; hs-CRP—high sensitivity C reactive protein; ICAM—intercellular adhesion molecule; IL—interleukin; NLRP3—NLR family pyrin domain containing 3; CV—cardiovascular, VCAM—vascular cell adhesion molecule; TNF—tumour necrosis factor; TGF—transforming growth factor.

Atherosclerosis is a complex pathology involving lipid metabolism, inflammation, and endothelial dysfunction [16]. Several of these mechanisms, identified in the pathogenesis of atherosclerosis, have been assessed in relation to SGLT2 inhibitors (Figure 1).

Lipid uptake into the sub-endothelium and formation of foam cells is one of the early processes in atherosclerotic plaque formation [16]. The importance of inflammation in atherosclerosis is also well established [17], not only in the development of atherosclerotic plaque, but also in precipitating acute ASCVD events. T2D is an inflammatory state and many studies have demonstrated that inflammation and oxidative stress are major factors leading to atherosclerosis development in these patients [18]. Monocyte recruitment, activation and differentiation, macrophage polarisation, and inflammasome activation contribute to atherosclerotic plaque formation and vulnerability [17,19,20]. Further, inflammatory cell content of plaque and cytokine activation and release are also established in the pathogenesis of atherosclerosis [17,21].

The endothelium is the major regulator of arterial homeostasis, including regulation of smooth muscle cell proliferation, cell migration, vascular reactivity, inflammation, and thrombosis through a number of mediators of which nitric oxide (NO) has a significant role [22]. Endothelial dysfunction is considered an early process in atherosclerosis, evident before clinical atherosclerotic plaque in arteries [23]. Smooth muscle cell proliferation and migration into denuded endothelium with injury, along with increased endothelial reactivity and altered cell adhesion molecule expression are well known in the pathogenesis of atherosclerosis and resultant ASCVD events [24]. Endothelial dysfunction is present in T2D and results in vascular inflammation and impaired vasorelaxation. The major factors contributing to endothelial dysfunction in T2D are hyperglycaemia, insulin resistance and the metabolic syndrome. These factors lead to increased vascular reactive oxygen species (ROS), impaired NO synthesis and degradation [22] and a prothrombotic tendency as well as changes to chemokines and direct mitochondrial oxidative stress [25].

## 4. Effects of SGLT2 Inhibitors on Atherosclerosis Pathways

### 4.1. Glycaemia

Atherosclerosis is driven by the uptake of lipids into the sub-endothelium, monocyte migration, and differentiation of macrophages into foam cells [26]. In vitro, high glucose levels have a detrimental effect on lipid metabolism and enhance foam cell formation promoting atherosclerosis [27,28]. It remains unclear, however, if hyperglycaemia and/or other mechanisms, result in foam cell accumulation and accelerated atherosclerosis in T2D [29]. Furthermore, diabetes has been shown to induce foam cell formation directly through increased lectin-like oxidized LDL receptor (LOX-1) and Class A scavenger receptors on macrophages in hyperglycaemic environments [27,28], and to accelerate the course of atherosclerotic disease. SGLT2 inhibitors modestly reduce serum glucose through glycosuria [30], which may be mechanistically linked with the observed reduction in ASCVD events seen with this drug class. Improved glycaemic control as a mechanism of reducing CV events has also been shown in recent studies of GLP-1 agonists [31]. However, several other glucose lowering agents, including sulfonylureas, thiazolidinediones, and insulin, do not reduce CV events [32], despite clear evidence that hyperglycaemia increases the risk of ASCVD events [33,34].

In addition to glucose lowering, SGLT2 inhibitors have also been shown to have effects on insulin resistance in both mouse and human studies [35,36]. Insulin resistance is strongly linked with atherosclerosis progression irrespective of hyperglycaemia [37]. Insulin resistance is pro-inflammatory and results in endothelial dysfunction, inflammatory cell entry into plaque, and promotes plaque vulnerability [38]. A reduction in aortic arch atherosclerotic plaque was demonstrated in diabetic ApoE^−/−^ knockout mice administered empagliflozin. These mice demonstrated metabolic changes of reduced body fat and weight in the empagliflozin group, as has been seen in clinical studies. Independent of body weight, atherosclerotic plaque and insulin resistance measured through HOMA-IR and fasting insulin levels were reduced in the empagliflozin group, compared to mice treated with glimepiride [39]. This improved insulin sensitivity with SGLT2 inhibition has been demonstrated in several other small human studies [40,41,42]. Thus, reduced insulin resistance has been proposed as a possible mechanism contributing to reduced atherosclerosis progression afforded by SGLT2 inhibitors. There is however conflicting evidence, with no increase in peripheral tissue insulin sensitivity in a small human clinical trial of dapagliflozin as measured by PET despite improved glycaemic control in a comparison against placebo with existing metformin and DPP4 inhibitor therapy [43]. The lack of ASCVD benefits seen with glimepiride treatment [39], which is also known to improve insulin sensitivity and is a more potent oral hypoglycaemic, alongside minimal difference in HbA1c between groups in CV outcome trials of SGLT2 inhibitors, suggest that glucose lowering and reduction in glucose mediated toxicity and insulin sensitivity may not be the only mechanism by which SGLT2 inhibitors afford ASCVD benefits [1,2]. 

Available evidence to date, therefore, does not conclusively elucidate the importance of SGLT2 inhibitor mediated glycaemic and insulin effects in reducing ASCVD events. 

### 4.2. Lipid Metabolism

Al Sharea et al. explored SGLT2 inhibitor effects on lipoprotein levels and atherosclerosis in a rodent model. They demonstrated significantly elevated atherogenic blood lipid profile and increased lipid and macrophage content of atherosclerotic plaque with induction of diabetes [44]. Maintenance of normoglycaemia with SGLT2 inhibitors significantly decreased lipid levels without affecting insulin levels [44] and reduced atheroma in aortas of diabetic mice, but not in nondiabetic mice. These benefits were thought to be mediated by lipoprotein clearance by the liver, defective in hyperglycaemic states [44]. However, other studies in rodent models are conflicting regarding lipid metabolism, demonstrating unchanged lipid profiles with SGLT2 inhibitor use [29,39,45].

Human studies have also failed to demonstrate consistent lipid benefits from SGLT2 inhibition with no change in LDL or triglycerides with empagliflozin treatment [46] and several recent meta-analyses demonstrating heterogeneity in results including some reporting no difference in lipids [47], and others an increase in high-density lipoprotein (HDL), LDL, and reduced triglycerides (TG) [48,49]. Furthermore, whilst the clinical benefits appear to be broadly consistent across the drug class, there is considerable heterogeneity across SGLT2 inhibitor types with respect to lipid lowering effects [49]. Therefore, it is unlikely that alterations in lipid metabolism are the primary mechanisms by which SGLT2 inhibitors reduce ASCVD events. 

### 4.3. Plaque Volume and Characteristics

The effect of SGLT2 inhibitors on hyperglycaemia, insulin resistance, foam cell formation, and cholesterol uptake have all been evaluated in animal models to inform a growing understanding of mechanisms linking SGLT2 inhibitors to reduced ASCVD events. A rodent model of T2D in atherosclerosis-prone mice demonstrated a reduction in both plasma glucose and atherosclerotic lesion size in the aorta with dapagliflozin, potentially mediated by a reduction in macrophage infiltration, and foam cell formation [29]. These findings have been confirmed in several T2D rodent models with different SGLT2 inhibitors [39,45], suggesting a role for SGLT2 inhibitors in promoting plaque regression. However, evidence for these effects in the absence of T2D are less clear. Conflicting data have been obtained in two small animal studies of the SGLT2 inhibitor dapagliflozin, in Apo E^−/−^ mice without T2D [29,44]. The first study, which demonstrated a reduction in atheroma, had a longer duration of therapy (12 compared to 4 weeks) than the second study, potentially accounting for the observed difference in efficacy [50]. In all studies, significantly more atheroma was present in diabetic mice compared to nondiabetic mice prior to SGLT2 inhibitor treatment; thus, the power to detect a significant reduction in atheroma in T2D mice may be greater. Furthermore, a correlation of HBA1c with foam cell formation, and foam cell formation with atherosclerosis, was only seen in diabetic mice. This correlation may be potentially confounded by limited power due to the very low HBA1c levels and lower numbers of foam cells and atherogenesis in non-diabetic mice. The mechanism of benefit of SGLT2 inhibitors may involve glucose metabolism and/or lipid uptake to macrophages in a deranged glycaemic environment, but a glucose independent mechanism is not excluded, given the benefits seen in some studies of non-T2D rodents and in non-diabetic human clinical trials.

Taken together, it remains unclear whether alterations in glucose and lipid metabolism are responsible for the reduced incidence of ASCVD events in those treated with SGLT2 inhibitors. 

## 5. Effects of SGLT2 Inhibitors on Inflammation 

The effects of SGLT2 inhibitors on athero-inflammation have been investigated in animal and human models. Reduced inflammatory cell infiltration in plaque has been demonstrated with reduced macrophage staining in aortic plaque of diabetic mice treated with SGLT2 inhibitors [39,45,51]. For example, empagliflozin reduced TNF-α, IL-6, and MCP-1 mRNA in aortas of ApoE^−/−^ mice compared to controls and glimepiride treated mice, after just 6–8 weeks of treatment [39]. Treatment with luseogliflozin and canagliflozin reduced aortic gene expression of adhesion molecules, metalloproteinases MMP-2 and MMP-9, the inflammatory cytokines TNF-α and IL-1 and 6, and MCP-1 in ApoE^−/−^ mice with induced diabetes, to levels comparable to non-diabetic ApoE^−/−^ mice [45,51], as well as reducing plaque burden in diabetic Apo E^−/−^ mice compared to controls [45]. These inflammatory cytokines and metalloproteinases are increased in unstable atherosclerotic plaque, suggesting a benefit of SGLT2 inhibitors in plaque stabilisation [45].

SGLT2 inhibitors also reduce circulating inflammatory cytokines in both mice and humans. For example, hs-CRP, TNF-α, IL-6, and MCP-1 serum levels all reduced after administration of empagliflozin and canagliflozin in diabetic mice [18,39,45,51]. Attenuated levels of circulating TNF-α have also been shown in non-diabetic, high fat diet obese mice (C57BL/6J) administered empagliflozin [39]. Human studies support these animal models showing a reduction in serum TNF-α, hs-CRP, IL-6, TGFβ, ferritin, and leptin in diabetic patients treated with SGLT2 inhibitors [46,52,53,54].

The NLRP3 Inflammasome is a multiprotein signalling complex found in monocytes and macrophages and is an important part of the innate inflammatory cascade [20,55]. Activation of the NLRP3 inflammasome results in inflammatory cytokine release including IL-18 and IL-1β, which are raised in ACS patients, and those with elevated CV risk [56,57]. Free fatty acids and elevated blood glucose has been shown to activate the inflammasome in T2D [50]. Inhibition of NLRP3 inflammasome activation with SGLT2 inhibitor treatment has been demonstrated in the kidney, and heart [58]. The mechanism of action includes inhibition of inflammasome priming via calcium dependent pathways, leading to a reduction in transcript levels of NLRP3, NF-kB, and caspase -1. Subsequent reduction in downstream IL-1β and IL-18 expression in cardiac tissue was also demonstrated. Reduced expression of these inflammatory cytokines persisted although the effect was blunted in the presence of calcium ionophores reflecting a calcium dependent mechanism or release [59]. Reduced NLRP3 activation has also been observed in an HFpEF model of rodents without T2D [59]. Furthermore, SGLT2 inhibition has been demonstrated to modulate inflammasome activity in small human trials in keeping with rodent models. A reduction in IL- 1β secretion from macrophages and reduction in transcript levels of NLRP3 and TNF-α has been shown confirming the mechanism of SGLT2 inhibitors to reduce NLRP3 activation in human macrophages [60]. Taken together, the demonstrated effects of NLRP3 attenuation in both T2D and non T2D rodent and human models suggest a glucose independent mechanism likely to contribute to the benefits seen in HF and MACE in human studies with SGLT2 inhibition.

A further mechanism of action may be effects on macrophage differentiation and infiltration. Differentiation of monocytes to macrophages with an M1 macrophage subtype polarization skewed in hyperglycaemic, hypoxic, and hyperlipidaemic states [61] results in secretion of pro-inflammatory cytokines driving plaque vulnerability [26,62]. SGLT2 inhibitor therapy has been shown to reduce macrophage infiltration and increase smooth muscle cell content in aortic atheroma of ApoE^−/−^ diabetic mice, with reduced plaque vulnerability via regulation of cellular infiltration [50]. Human studies assessing macrophage differentiation with SGLT2 inhibitor use, have also shown M1/M2 phenotype shift with SGLT2 inhibitors [63], suggesting a further cardioprotective mechanism of action [64]. 

Therefore, it is likely that favourable effects on inflammation are mechanistically important in the reduced ASCVD risk seen with SGLT2 inhibitor treatment.

## 6. Effects of SGLT2 Inhibitors on Endothelial Function

Smooth muscle cells play a key role in plaque stabilisation through forming a fibromuscular cap [16]. The effect of SGLT2 inhibitors on endothelial and smooth muscle cell proliferation has been investigated in rat aortic cells. These demonstrated no increase in endothelial and vascular smooth muscle cell (VSMC) proliferation with empagliflozin [39]. However a reduced expression of VCAM, a vascular endothelial cell adhesion molecule, with SGLT2 inhibitors, has been shown in ApoE^−/−^ mice [51,65,66]. Additionally, reduced superoxide production in the thoracic aorta and improved vasorelaxation in db/db mice with impaired endothelial function due to acetylcholine has also been demonstrated with SGLT2 inhibitor treatment [66]. Further demonstration of SGLT2 inhibitor induced vasorelaxation of VSMC has been shown in rabbit aortas in a concentration dependent manner [67]. Empagliflozin has also been shown in cultured human aortic VSMC’s to block proliferation and migration in a stimulated environment with IL-17A [68].

Vascular endothelial reactivity is also improved with SGLT2 inhibitor treatment. For example, microvascular function assessed by coronary flow velocity reserve, measured on echocardiography using isoflurane to induce maximal hyperaemia, has been shown to improve after 5 and 10 weeks of empagliflozin in insulin resistant obese C57BL/6J mice (ob/ob^−/−^) mice compared with age-matched lean and untreated ob/ob^−/−^ mice [69]. Aortic rings applied to mouse aortas in culture, in hyperglycaemic conditions, show severely impaired endothelial NO vasodilatation, corrected by SGLT2 inhibition [70]. Moreover, direct acetylcholine induced vasorelaxation in vivo has been demonstrated with dapagliflozin, and to a greater extent in denuded endothelium in non-diabetic ApoE^−/−^ mice, suggesting a possible complex mechanism of action on endothelial function, a known early step in atherosclerosis [71].

In vitro studies using human umbilical vein endothelial cells (HUVEC’s) to assess endothelial cell proliferation and adhesion molecule expression, alongside vessel vasodilatation through flow-mediated dilatation and neointimal hyperplasia have been assessed in the context of SGLT2 inhibitor use. These studies have shown no difference in proliferation of VEGF stimulated HUVEC’s with SGLT2 inhibitor administration [39], suggesting no role of SGLT2 inhibitors in endothelial cell proliferation. However, vascular endothelial cell responses to SGLT2 inhibitors, assessed by Gaspari et al. demonstrated attenuated cell adhesion molecule expression in HUVEC’s stimulated with TNF-α in the setting of hyperglycaemia and attenuated ICAM expression in a hyperglycaemic environment without stimulation [71]. There was no attenuation of ICAM or VCAM protein expression in non-stimulated HUVECS with SGLT2 inhibitor dapagliflozin suggesting SGLT2 inhibitors may act on endothelium through adhesion molecule regulation on the endothelium. Empagliflozin has also been demonstrated to prevent cell death in HUVEC’s exposed to hypoxic stress in culture and reduce infarct size after ischaemia/reperfusion injury in mice, suggesting SGLT2 inhibitors reduce the impact of oxidative stress [72]. In vitro studies of antioxidant effect of SGLT2 inhibitors on human coronary artery endothelial cells (HCAEC’s) similarly demonstrated reduced cell permeability and reactive oxygen species production compared to control [73]. Clinical studies assessing flow mediated dilatation (FMD) of the brachial artery, a surrogate for endothelial dysfunction in coronary arteries and systemically [23], demonstrated improved changes in FMD from baseline with SGLT2 inhibitors compared to metformin at 16 weeks in those with early stage diabetes [74]. 

A reduction in neointimal hyperplasia with SGLT2 inhibitor administration is a further proposed mechanism of action on the endothelium by SGLT2 inhibitors. Neointimal thickness of coronary arteries has been assessed post bioresorbable polymer drug eluting stent implantation for coronary stenosis in a human study, assessing ACS and stable angina populations by optical coherence tomography (OCT). This demonstrated a reduction in neointimal hyperplasia in patients treated with SGLT2 inhibitors versus other oral hypoglycaemic agents 1 year after initiation. Body weight and blood pressure were significantly associated with neointimal hyperplasia changes, but not with blood glucose measurement [75]. Similarly, neointimal hyperplasia reduction with SGLT2 inhibition in injured femoral arteries of high fat diet mice has also been demonstrated [76]. SGLT2 inhibitors have also been shown to improve endothelial function and aortic stiffness in humans as measured by central systolic pressure, pulse wave velocity (PWV) [77,78], renal resistance index, and FMD of the brachial artery [79].

Taken together, there is preliminary evidence that SGLT2 inhibitors have positive effects of vascular reactivity, oxidative stress, and plaque stability.

## 7. Limitations and Future Directions

A key weakness of the data from many of these mechanistic studies is that the majority of the work has been done in diabetic models of disease. Further, many have showed mechanisms of action and disease benefits that are restricted to diabetic models and not observed outside of diabetes. This is clearly inconsistent with the broader clinical benefits seen in those with HF and CKD irrespective of the presence of diabetes and raises significant uncertainty about much of the mechanistic research underpinning our understanding of how SGLT2 inhibitors drive clinical benefit.

Large human studies with mechanistic endpoints assessing the production and release of inflammatory cytokines, detailed effects on lipid metabolism, the impact on endothelial function and diverse measures of atherosclerosis burden have significant potential to add to our understanding of the mechanisms underpinning the clinical benefits of SGLT2 inhibitors for ASCVD events.

## 8. Conclusions

SGLT2 inhibitors have emerged as a class of drugs with broad cardiovascular benefits that extend well beyond the initial target population of individuals with T2D. There are clear and comparable benefits in those with CKD and HF regardless of the presence of T2D. These benefits include reductions in MACE and CV death. The mechanisms underpinning the observed benefits for ASCVD remain uncertain, but are clearly not the sole result, or the primary consequence, of a mechanism dependent upon modifying aberrant blood glucose levels, as was hypothesised during the early development of this drug class.

The combined human and animal data suggest multiple possible pathways mediated by not only effects on glucose management, but also pathways moderated by lipid metabolism and foam cell formation in the sub-endothelium, inflammation, and endothelial function.

Whilst the beneficial effects of SGLT2 inhibitors on established intermediate markers of cardiometabolic health, such as blood pressure and body weight, are clear, these changes are unlikely to fully explain the ASCVD benefits seen. Likewise, the absence of stroke protection, despite clear blood pressure lowering, is unexplained, and suggest undiscovered effects of SGLT2 on this outcome.

## Figures and Tables

**Table 1:** SGLT2 inhibitors main cardiovascular outcome trials summary.

Completed Trial	Intervention	Study Size (n)	CV Disease Proportion, n (%)	Primary Outcome	MACE, HR (95% CI)	MI ^1^, HR (95% CI)	Stroke ^1^, HR (95% CI)	CV Mortality, HR (95% CI)
EMPA-REG OUTCOME [2]	empagliflozin	7020	6964 (99.2) ^2^	MACE	0.86 (0.74–0.99)	0.87 (0.70–1.09)	1.18 (0.89–1.56)	0.62 (0.49–0.77)
CANVAS Program [1]	canagliflozin	10142	6656 (65.6) ^2^	MACE	0.86 (0.75–0.97)	0.89 (0.73–1.09)	0.87 (0.69–1.09)	0.87 (0.72–1.06)
DECLARE-TIMI 58 [3]	dapagliflozin	17160	6974 (40.6) ^2^	MACE	0.93 (0.84–1.03)	0.89 (0.77–1.01)	1.01 (0.84–1.21)	0.98 (0.82–1.17)
CREDENCE [4]	canagliflozin	4401	2220 (55.4) ^2^	Composite of ESKD, doubling of serum creatinine, renal, or CV death	0.80 (0.67–0.95)	0.86 (0.64–1.06)	0.77 (0.55–1.08)	0.78 (0.61–1.00)
DAPA-HF [10]	dapagliflozin	4744	2674 (56.4) ^3^	Worsening HF (hospitalization or an urgent visit resulting in intravenous therapy for HF) or CV death	NA	NA	NA	0.82 (0.69–0.98)
VERTIS-CV [8]	Ertugliflozin	8246	8236 (99.9) ^2^ [14]	MACE	0.97 (0.85–1.11)	1.04 (0.86–1.26)	1.06 (0.82–1.37)	0.92 (0.77–1.11)
DAPA-CKD [9]	dapagliflozin	4304	1610 (37.4) ^4^	Composite of ≥ 50% sustained decline in eGFR, ESKD, renal, or CV death	NA	NA	NA	0.81 (0.58–1.12)
EMPEROR-Reduced [11]	empagliflozin	3730	1929 (51.7) ^3^	CV death or hospitalization for worsening HF	NA	NA	NA	0.92 (0.75–1.12)
SOLOIST-WHF [15]	sotagliflozin	1222	NA	CV death and hospitalizations and urgent visits for HF	NA	NA	NA	0.84 (0.58–1.22)
SCORED [7]	sotagliflozin	10,584	5144 (48.6) ^5^	CV death, hospitalizations for HF, and urgent visits for HF	0.84 (0.72–0.99)	0.68 (0.52–0.89)	0.66 (0.48–0.91)	0.90 (0.73–1.12)

MACE: major adverse cardiovascular events, a composite of death from cardiovascular causes, nonfatal myocardial infarction, or nonfatal stroke; MI: myocardial infarction; HR: hazard ratio; CV: cardiovascular; HF: heart failure; ESKD: end-stage kidney disease (dialysis, transplantation, or a sustained estimated GFR of < 15 mL per minute per 1.73 m^2^). ^1^ including fatal or nonfatal. ^2^ cardiovascular disease was defined as a history of coronary artery disease, cerebrovascular disease or peripheral artery disease. ^3^ numbers of patients with ischemic cardiomyopathy in EMPEROR-Reduced or DAPA-HF trial. ^4^ Cardiovascular disease was defined as a history of peripheral artery disease, angina pectoris, myocardial infarction, percutaneous coronary intervention, coronary-artery bypass grafting, heart failure, valvular heart disease, abdominal aorta aneurysm, atrial fibrillation, atrial flutter, ischemic stroke, transient ischemic attack, haemorrhagic stroke, carotid artery stenosis, cardiac-pacemaker insertion, vascular stent, coronary-artery stenosis, ventricular arrhythmia, implantable cardioverter–defibrillator, noncoronary revascularization, or surgical amputation. ^5^ History of myocardial infarction, stroke, coronary revascularization, or peripheral vascular disease (documented PAD, peripheral revascularization, or peripheral venous disease).
